# Recognition of Pep-13/25 MAMPs of *Phytophthora* localizes to an *RLK* locus in *Solanum microdontum*

**DOI:** 10.3389/fpls.2022.1037030

**Published:** 2023-01-12

**Authors:** Xiao Lin, Yerisf Carla Torres Ascurra, Happyka Fillianti, Laura Dethier, Laura de Rond, Emmanouil Domazakis, Carolina Aguilera-Galvez, Afewerki Yohannes Kiros, Evert Jacobsen, Richard G. F. Visser, Thorsten Nürnberger, Vivianne G. A. A. Vleeshouwers

**Affiliations:** ^1^ Plant Breeding, Wageningen University and Research, Wageningen, Netherlands; ^2^ Department of Plant Biochemistry, Centre of Plant Molecular Biology (ZMBP), University of Tübingen, Tübingen, Germany; ^3^ Department of Biochemistry, University of Johannesburg, Johannesburg, South Africa

**Keywords:** Potato late blight, *Phytophthora infestans*, MAMP, RLK, Pep-13/25, BSR-Seq

## Abstract

Pattern-triggered immunity (PTI) in plants is mediated by cell surface-localized pattern recognition receptors (PRRs) upon perception of microbe-associated molecular pattern (MAMPs). MAMPs are conserved molecules across microbe species, or even kingdoms, and PRRs can confer broad-spectrum disease resistance. Pep-13/25 are well-characterized MAMPs in *Phytophthora* species, which are renowned devastating oomycete pathogens of potato and other plants, and for which genetic resistance is highly wanted. Pep-13/25 are derived from a 42 kDa transglutaminase GP42, but their cognate PRR has remained unknown. Here, we genetically mapped a novel surface immune receptor that recognizes Pep-25. By using effectoromics screening, we characterized the recognition spectrum of Pep-13/25 in diverse Solanaceae species. Response to Pep-13/25 was predominantly found in potato and related wild tuber-bearing Solanum species. Bulk-segregant RNA sequencing (BSR-Seq) and genetic mapping the response to Pep-25 led to a 0.081 cM region on the top of chromosome 3 in the wild potato species *Solanum microdontum* subsp. *gigantophyllum*. Some BAC clones in this region were isolated and sequenced, and we found the Pep-25 receptor locates in a complex receptor-like kinase (*RLK*) locus. This study is an important step toward the identification of the Pep-13/25 receptor, which can potentially lead to broad application in potato and various other hosts of *Phytophthora* species

## Introduction

Plants have evolved two layers of innate immune system to perceive non-self molecules and elicit immune responses. Plasma membrane-localized immune receptors, typically receptor-like proteins (RLPs) and receptor-like kinases (RLKs), form the first layer of defense. These surface receptors recognize microbial-associated molecular patterns (MAMPs) or apoplastic effectors and induce pattern-triggered immunity (PTI). Inside the plant cells, nucleotide-binding domain and leucine-rich repeat-containing receptors (NLRs) recognize pathogen intracellular effectors and mediate effector-triggered immunity (ETI) (Jones and Dangl, 2006).

*Phytophthora* species are renown as notorious plant pathogens that trigger pandemics in many important crops, like potato late blight, sudden oak death, soybean root rot, and tobacco black shank disease, caused by *Phytophthora infestans*, *P. ramorum*, *P. sojae* and *P. parasitica* respectively. Various MAMPs of oomycetes, such as Pep-13, nlp20, INF1, XEG1 PcF and CBEL (Ricci et al., 1989; Orsomando et al., 2001; Brunner et al., 2002; Liu et al., 2005; Gaulin et al., 2006; Ma et al., 2015) have been characterized. Yet for only a few oomycete PAMPS, the matching surface immune receptors have been cloned so far, namely ELR, RLP23 and RXEG1, that recognize INF1, nlp20 and XEG1 respectively (Albert et al., 2015; Du et al., 2015; Wang et al., 2018), all the three characterized PRRs are LRR-RLPs, and these PRRs have shown to enhance resistance to the respective oomycete pathogen. Remarkably, the structure of XEG1-BAK1-RXEG1 (LRR) complex was resolved recently, it revealed the mechanism of LRR-RLP activation upon ligand recognition (Sun et al., 2022).

Pep-13 is derived from GP42, a 42 kDa transglutaminase (TGase) that was firstly isolated from *Phytophthora sojae* culture filtrates (Parker et al., 1991). To find the minimal peptide of the elicitor, various endo- and exopeptidases were used to digest GP42, and a peptide of 13 amino acid residues (Pep-13) was found sufficient for the elicitor activity in parsley cell cultures (Nürnberger et al., 1994). Pep-25 is a longer peptide that shows a similar activity as Pep-13. Pep-13/25 are highly conserved among *Phytophthora* species. In parsley cell culture, Pep-13 induces defense responses including, oxidative burst, ion fluxes, phytoalexin formation, and defense-related gene activation (Nürnberger et al., 1994). In potato, infiltration of Pep-13 and Pep-25 into the leaves can induce hypersensitive response (HR) and expression of the defense-related genes (Brunner et al., 2002). The Pep-13 triggered immunity was reported to be SERK3A/B (BAK1)-dependent in potato (Nietzschmann et al., 2019). However, the Pep-13/25 receptor has not been cloned so far.

Map-based cloning is a traditional strategy for gene mapping and cloning, however it is time-consuming and laborious. With the fast evolving of next and third generations of sequencing technology, genetic mapping becomes easier and faster than ever before. For potato, many reference genomes are available now, like *Solanum tuberosum* group Phureja DM1-3 516 R44, RH89-039-16, Solyntus, *Solanum verrucosum*, *Solanum chacoense*, and recently a tetraploid potato cultivar was also assembled into chromosome level (Xu et al., 2011; Leisner et al., 2018; Paajanen et al., 2019; van Lieshout et al., 2020; Zhou et al., 2020; Sun et al., 2022). Additionally, target enrichment sequencing and bulk segregant analysis (BSA) like Resistance gene enrichment sequencing (RenSeq), RLP/K enrichment sequencing, and BSA-Seq can also help to clone new resistance genes (Witek et al., 2016; Zou et al., 2016; Lin et al., 2020a; Witek et al., 2021).

Here, we performed a large-scale infiltration of Pep-13 and Pep-25 peptides in different Solanaceae species. We found that Pep-13/25 recognition is relatively common in potato cultivars, less prevalent in wild potatoes, and absent in other Solanaceae families. Then, we generated a segregating population of Pep-25 responsiveness by several rounds of crossings of diverse diploid wild potato genotypes. By using BSR-Seq and map-based cloning strategy, the Pep-25 receptor was finally fine-mapped to a 0.081 cM *LRR*-*RLK* gene locus on the top of chromosome 3. Our findings will lead to the identification of the Pep-13/25 receptor.

## Materials and methods

### Peptide synthesis

Pep13 (VWNQPVRGFKVYE) and Pep25 (DVTAGAEVWNQ4PVRGFKYEQTEMTE) were synthesized by GenScript (USA). The peptides were dissolved in MQ to a concentration of 1-3 µM. The peptides Pep-13 and Pep-25 were infiltrated into the abaxial side of plant leaves by a needleless syringe, and the cell death phenotype was scored 3 days after infiltration.

### Plant materials

Seeds of 24 tomato, 7 eggplant, and 10 pepper accessions were obtained from the Centre for Genetic Resources, the Netherlands (CGN). They were sown in the greenhouse, and the peptides infiltration was performed 6 weeks after germination. In addition, seeds from *Nicotiana benthamiana*, *N. glutinosa*, and 6 cultivars of *Nicotiana tabacum*, including cv. Rustica, cv. White burley, cv. Cleveland, cv. Samsun, cv. Xanthii, cv. SR1 were obtained from Unifarm of Wageningen University and Research. Six weeks-old plants were used for peptides infiltration. Wild species and potato cultivars were obtained from the *in vitro* collection of Plant Breeding, Wageningen University and Research. These plants are maintained *in vitro* on MS20 medium at 25°C. Top shoots of plants were cut and clonally propagated 2 weeks before transfer to the soil. All plants were grown in a climate-controlled greenhouse compartment at 22°C/18°C day/night temperature regime under long-day conditions. In all cases, three leaves per plant, and three plants per accession were used.

### Mapping population

The crossings between selected lines were made in different years. Seedlings were propagated and grown *in vitro* on MS20 medium for 2 weeks, and then transferred to the greenhouse. The female parental line was emasculated before anthesis, and 3 days later was pollinated with the donor. Ripe berries were collected, and the seeds were recovered, cleaned with water, and air-dried on filter paper before packaging.

The seeds were sterilized before germination. They were rinsed in 70% ethanol and soaked in a solution of 1.5% hypochlorite and Tween 20, then they were washed with autoclaved water to remove the hypochlorite. For the recombinant screening, the seeds were sown on MS20 medium (with 1000 ppm GA3, if the seeds were new for breaking the dormancy) or in the greenhouse directly. For the plants in greenhouse, after genotyping, the selected recombinants were sterilized and moved into the *in vitro* collection for later analyses.

### Sample preparation and RNA isolation for BSR-Seq

One hundred seeds from population 3521 were sown in the greenhouse, when they were 6 weeks old, Pep-25 peptides and water (negative control) were infiltrated for phenotyping. The infiltration was performed on 3 leaves and was repeated at least 3 times on the same plant. The cell death phenotype was scored 3-4 days after infiltration, then the leaves from 34 Pep-25 responding (R bulk) and 34 Pep-25 non-responsive progenies (NR bulk), as well as the parental line 3341-15 were collected for inoculation with *P. infestans*. Forty-eight hours after inoculation, two 1 cm-leaf discs from the same plant were collected into 2 ml tubes containing 2 small metal beads and immediately frozen with liquid nitrogen. Six samples were collected in total, i.e. R bulk mock, R bulk inoculated with *P. infestans* isolate Dinteloord, NR bulk mock, NR inoculated with *P. infestans* isolate Dinteloord, 3341-15 mock, and 3341-15 inoculated with *P. infestans* Dinteloord. The 6 samples were ground by TissueLyser II (QIAGEN). Then 100 mg samples were used for RNA isolation by Rneasy Plus Mini Kit from QIAGEN following industrial instructions. The gDNA eliminator spin column from the kit was used to remove the gDNA. The six RNA samples were tested by agarose electrophoresis, quantified by Nanodrop (ThermoFisher) and sent to Novogene (Beijing, China) with dry ice for RNA sequencing. The RNAseq data from Pep-25 non-responsive parent MCD360-1 were obtained from previous studies (Lin et al., 2020b).

### Bioinformatic analysis

Paired-end Illumina HiSeq reads were first checked with FastQC (v0.10.0; http://www.bioinformatics.babraham.ac.uk/projects/fastqc/) and the adapters were trimmed with trimmomatic v0.36 (Bolger et al., 2014). The trimmed reads were then mapped to the potato DM reference genome (v4.03) using STAR v2.5 (Dobin et al., 2013). Pileup files were generated for the bulk and parents using SAMtools mpileup with default settings and piped into VarScan mpileup2snp (v2.3.7) (Koboldt et al., 2012). SNPs were filtered using a custom Java code (Lin et al., 2020a) to retain informative SNPs present in both bulks and both parents. SNPs were filtered based on expected allele ratios in responsive (Rr)/non-responsive (rr) samples. To be retained, each SNP had a minimum read depth of 50 and alternate allele ratios reflecting the expected genotype: 0-10% or 90-100% alternate allele for rr and 40-60% alternate allele for Rr. BEDTools intersect (v2.20.1) (Quinlan and Hall, 2010) was used to extract SNPs present in both bulks and parents (informative SNPs) and to relate the informative SNP locations to transcripts of the reference genome. The number of SNPs were plotted in 1 Mb bins across each chromosome and visualized using R (Lin et al., 2020a).

### High resolution melting (HRM) marker development and analysis

The BAM and VCF files (filtered informative SNPs) were imported into Geneious R10 for visualization (Kearse et al., 2012) (http://www.geneious.com). The primers were designed in Geneious R10, ideally, the PCR product should only contain one informative SNP and the size should be between 80-150bp. Primers flanking the informative SNPs were manually selected on the conserved sequences of both parents, R and NR bulks. The protocols for DNA isolation and HRM markers were described previously (Lin et al., 2020a).

### BAC library screening

The BAC library of GIG362-6 was generated as described previously (Lin et al., 2020a). PCR primers were designed in the mapping interval based on the DM genome for detecting the positive BAC clones.

### Candidate gene cloning and agroinfiltration

The coding region of the candidate genes was PCR-amplified and firstly cloned into the Gateway entry vector, then shuffled into destination vector pK7WG2 with 35S promoter. Then the constructs were transformed into *Agrobacterium tumefaciens* strain AGL1 for transiently overexpression assay in plants. The agroinfiltration was performed in 4 –weeks-old *N. benthamiana* leaves, Pep-25 peptides (2 μM) were infiltrated into the same leaves two days later.

### *P. infestans* inoculation

*P. infestans* isolate Dinteloord were propagated on rye medium for 14 days in a climate chamber (18°C). The zoospores were collected in cold water, 10 μL zoospore suspension (5 x 10^4^ zoospores/ml) was used to inoculate detached leaves. The leaves were sampled from 10 weeks-old Pep-25 responsive and non-responding progenies of population 3341-15 x MCD360-1.

### Phylogenetic analysis

The kinase domains of 365 potato RLK proteins and 12 candidate RLK proteins from GIG362-6 were included (Lin et al., 2020a). I3 from tomato was used as an outgroup, 21 known RLK proteins were also included as reference. The kinase domains were aligned in Geneious (Kearse et al., 2012), Maximum likelihood (ML) tree was made by iqtree (v1.6.10), LG+F+R7 was selected as the best-fit model, 1000 samples were generated for the ultrafast bootstrap analysis (Kalyaanamoorthy et al., 2017; Hoang et al., 2018; Minh et al., 2020).

### Data availability

The raw sequencing data were deposited in GenBank SRA under project number PRJNA893349. The BAC sequence was deposited in GenBank (OP716690).

## Results

### Pep-13/25 trigger HR on cultivated and wild potatoes

Pep-13/25 are conserved regions in the cell wall-associated, Ca^2+^-dependent transglutaminase (GP42), the structure of GP42 (PDF code 3TW5) from *Phytophthora sojae* was solved (Reiss et al., 2011), and is visualized in **Figure S1A**, Pep-13 peptides are highlighted by cyan. Pep-13 and Pep-25 were found among ten *Phytophthora* species (Brunner et al., 2002). The Pep-13 and Pep-25 sequences from *P. infestans*, *P. sojae*, *P. palmivora*, *P. parasitica*, *P. cactorum*, *P. ramorum*, *P. cinnamomi*, and *P. capsici* are visualized in **Figure S1B**.

**Figure 1:**
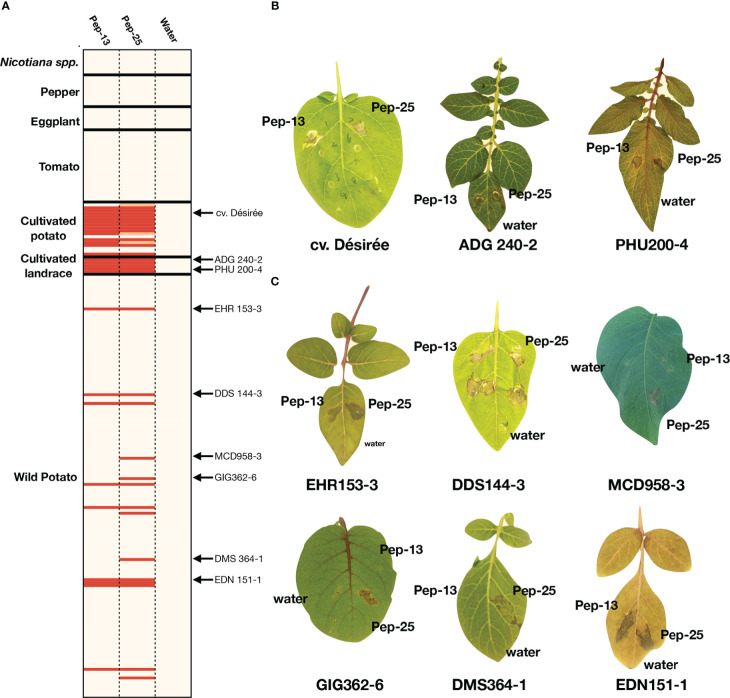
Screening for Pep-13/25 recognition in multiple Solanaceae species **(A)**. Heatmap of Pep-13 and Pep-25 recognition in different Solanaceae species and genotypes. The HR severity was scored. Strong HRs after Pep-13/25 infiltration are highlighted by red, weak HRs are highlighted by orange, beige indicates no HR. The genotypes with arrows are shown in **(B, C)**. The detailed scoring table is shown in **Supplementary Table 1**. **(B)**. Examples of Pep-13/25 recognition in cultivated potatoes and landraces. *Solanum tuberosum* Group tuberosum cv Désirée, *S. tuberosum* Group andigena (ADG 240-2) and *S. phureja* (PHU200-4) respond to both Pep-13 and Pep-25. **(C)** Examples of Pep-13/25 recognition in wild *Solanum* spp. Genotypes of *S. ehrenbergii* (EHR), *S. doddsii* (DDS), *S. microdontum* (MCD), *S. microdontum* subsp. *gigantophyllum* (GIG), *S. demissum* (DMS) and *S. edinense* (EDN) are shown. EHR153-3, DDS144-3 and EDN151-1 respond to both Pep-13 and Pep-25, but MCD958-3, GIG362-6, DMS364-1 respond only to Pep-25.

Pep-13 was reported to elicit defense responses or cell death in parsley and potato cv. Désirée (Hahlbrock et al., 1995; Halim et al., 2004). However, whether both Pep-13/25 could be widely recognized in different plant families remains unknown. Here, we performed a large-scale screening of a collection of Solanaceae plants by Pep-13 and Pep-25 peptides infiltration. Firstly, we tested 19 potato cultivars, including the previous reported potato cultivar Désirée as a positive control, and some progenitor species of cultivated potato, i.e., *S. tuberosum andigena* (ADG240-2), PHU372-8 and PHU200-4, *S. stenotomum* (STN829-3), and *S. candolleanum* (CND531-3). We found Pep-13/25 recognition is common in potato cultivars, thirteen out of nineteen potato cultivars recognize both Pep-13/25, and five do not recognize any, or only show a weaker cell death phenotype to Pep-25 (**Figure 1A**). All five tested landraces recognize both Pep-13/25, however, the proposed progenitor of cultivated potato *S. candolleanum* (CND531-3) does not (**Figure 1B**).

To test if Pep-13/25 can trigger cell death in wild potatoes, peptide infiltration was performed in 146 wild potato genotypes belonging to 56 species. Among the 146 genotypes, 14 genotypes that belong to at least 8 different tuber-bearing *Solanum* species/sub-species showed cell death after infiltration of Pep-13 and/or Pep-25 (**Figures 1B, C**, **Table S1**). These 8 wild potato species/sub-species include *Solanum doddsii* (DDS), *Solanum demissum* (DMS), *Solanum edinense* (EDN), *Solanum hondelmannii* (HDM), *Solanum leptophyes* (LPH), *Solanum microdontum* (MCD), *Solanum microdontum* subsp. *gigantophyllum* (GIG), *Solanum chacoense* (CHC), *Solanum ehrenbergii* (I), and two unclassified species (SPEC) (Jacobs et al., 2008). We found that 36% of the responsive wild potatoes only showed response to Pep-25, but not Pep-13, which may be explained by a lower stability of Pep-13 in the harsh conditions in the apoplast, or, could point to other receptors in potatoes with different recognition specificity to Pep-13 versus Pep-25

To further determine whether Pep-13 and Pep-25 recognition is common in other members of the Solanaceae family, we screened the peptides in twenty-four tomato accessions, seven eggplant accessions, ten pepper accessions, and eight *Nicotiana* accessions. However, none of them recognize either Pep-13 or Pep-25 (**Figure 1**; **Table S1**). These results indicate Pep-13/25 recognition might be limited to a subset of *Solanum* species.

### Genetic mapping of Pep-25 receptor by BSR-Seq

**Figure 2:**
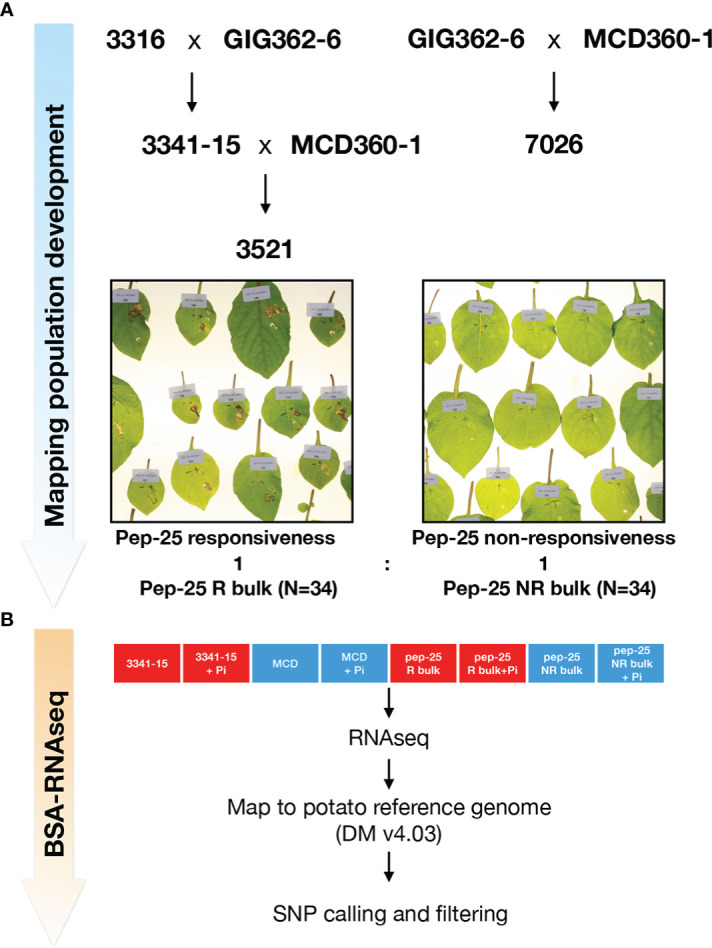
Mapping Pep-25 receptor to the top of Chromosome 3 by BSR-Seq **(A)** Mapping population development for mapping Pep-25 receptor. *S. microdontum* subsp. *gigantophyllum* GIG362-6 (R) was crossed with *S. verrucosum* 3316-17 (NR) and all progenies of the F1 population (3341) respond to Pep-25. Then, the segregating population 3521 was generated by crossing 3341-15 (R) with *S. microdontum* MCD360-1 (NR). The 34 R and 34 NR progenies were bulked for the BSR-Seq. R, respond to Pep-25; NR, not respond to Pep-25 **(B)** Two parental lines 3341-15 and MCD360-1, and two bulks (Pep-25 R and NR bulks) were treated with water (mock) or *Phytophthora infestans* zoospores followed by RNA extraction and RNA-seq. The reads were mapped to the potato reference genome (DMv403), and the SNPs were called, the informative SNPs were filtered.

Although the responses of Pep-13 and Pep-25 in plants were well characterized, the corresponding plant receptor remains unknown. The Pep-13/25 screening has revealed that there is genetic variation for response to Pep-13 and/or Pep-25. For instance, *Solanum microdontum* subsp. *gigantophyllum* (GIG362-6), a diploid species that responds to Pep-25, but not Pep-13; and *Solanum microdontum* (MCD360-1), which shows no response to Pep-13/25. To generate a mapping population of Pep-25, population 7026 was generated by crossing GIG362-6 and MCD360-1, and subsequently tested for segregation of Pep-25 response (**Figure 2A**). However, all the F1 progenies of 7026 respond to Pep-25 after peptides infiltration. Similarly, crossing GIG362-6 with the Pep-13/25 non-responsive *Solanum verrucosum* 3316-17, also led to a population (3341) in which all F1 progeny plants respond to Pep-25 (**Figure 2A**). These data indicate that the Pep-25 locus is likely homozygous in GIG362-6.

To generate a segregating population of Pep-25 response, we crossed the responsive 3341-15 with the non-responsive MCD360-1 to generate population 3521. One hundred and ten progeny plants of population 3521 were tested, 49 of them were responsive to Pep-25, 61 were non-responsive. This segregating ratio is close to 1:1 (χ2 p=0.252), suggesting that the Pep-25 response is determined by a single dominant gene that is homozygous in GIG362-6.

To genetically map the potential Pep-25 receptor, 34 Pep-25 responsive and 34 Pep-25 non-responsive individuals from population 3521 were pooled for BSR-Seq (**Figure 2A**). The parental lines 3341-15 and MCD360-1 were included for the RNAseq, to allow identification of high-quality SNPs in the downstream bioinformatics analysis. In addition, we included samples with *P. infestans* infection and mock control (water) to be able to explore up-regulated defense-related genes. The BSR-Seq bioinformatics pipeline are shown in **Figure 2B**.

### Fine mapping of Pep-25 receptor to an *RLK* gene locus on chromosome 3

**Figure 3:**
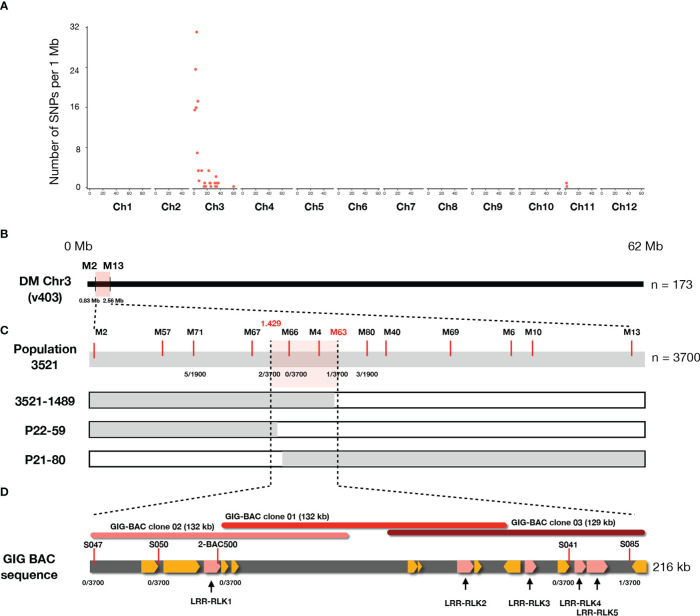
The Pep-25 receptor is fine-mapped to an *RLK* gene locus in *Solanum microdontum*. **(A)** The number of filtered SNPs per 1 Mb interval are visualized (*y* axis), the chromosome IDs are shown in *x* axis. Most of the informative SNPs are located on the top of chromosome 3, based on the potato reference genome (DM v4.03). **(B)** The Pep-25 receptor is mapped to a 1.73 Mb interval, between markers M2 and M13. **(C)** Fine-mapping of Pep-25 receptor in population 3521 (n=3700) to a 0.081 cM interval, between markers 1.429 and S085. Markers are shown with red lines, markers M66 and M4 are co-segregating with the Pep-25 response, the number of recombination/total plants are shown below the markers. **(D)**. A 216 kb contig generated from three BAC clones (01-03) from GIG362-6 shows 15 predicted *RLK* genes (highlighted yellow), five of these *RLK* are up-regulated upon *P. infestans* infection (highlighted by pink).

To identify the causal SNPs of Pep-25 responsiveness, the BSR-Seq reads were mapped to the potato reference genome (DMv403), and SNPs were called and filtered. The most informative SNPs linked to the Pep-25 responsiveness are located on the top of Chromosome 3 (**Figure 3A** and **Table S2**). To verify these informative SNPs, we designed high-resolution melting (HRM) markers and firstly tested them in a small population (n=173). We obtained two flanking markers (M2 and M13) and located the candidate Pep-25 receptor in a 1.73 Mb region (**Figure 3B**).

Then we developed a rapid-genotyping pipeline. In brief, the seeds were sown *in-vitro* and grown in MS20 medium, some leaf samples were cut from the *in-vitro* plants and used for DNA isolation, then the two flanking markers were used for genotyping. The recombinants were maintained *in-vitro* and propagated for phenotyping of Pep-25 response (Torres Ascurra et al., 2021). Molecular markers were designed based on the potato reference genome DM v4.03 (**Figure S3** and **Table S2**). In total, 3700 progenies individual were genotyped, and three recombinants were identified, which allowed us to fine-map Pep-25 receptor to a small interval between markers 1.429 and M63 (1.528) (**Figures 3C**; **S3** and **Table S2**). The genetic distance of this interval is ~0.081 cM (3/3700).

To obtain the physical map of this region, three BAC clones were isolated from a BAC library of GIG362-6 (Lin et al., 2020b), and they were sequenced. These three BAC clones cover 216 kb region, marker S085 locates at the end of the BAC sequence (**Figure 3D**), however, all other markers (S047, S050, 2-BAC500, S041) are co-segregating with Pep-25 responsiveness (**Figure 3D**).

### Candidate genes of Pep-25 receptor

**Figure 4:**
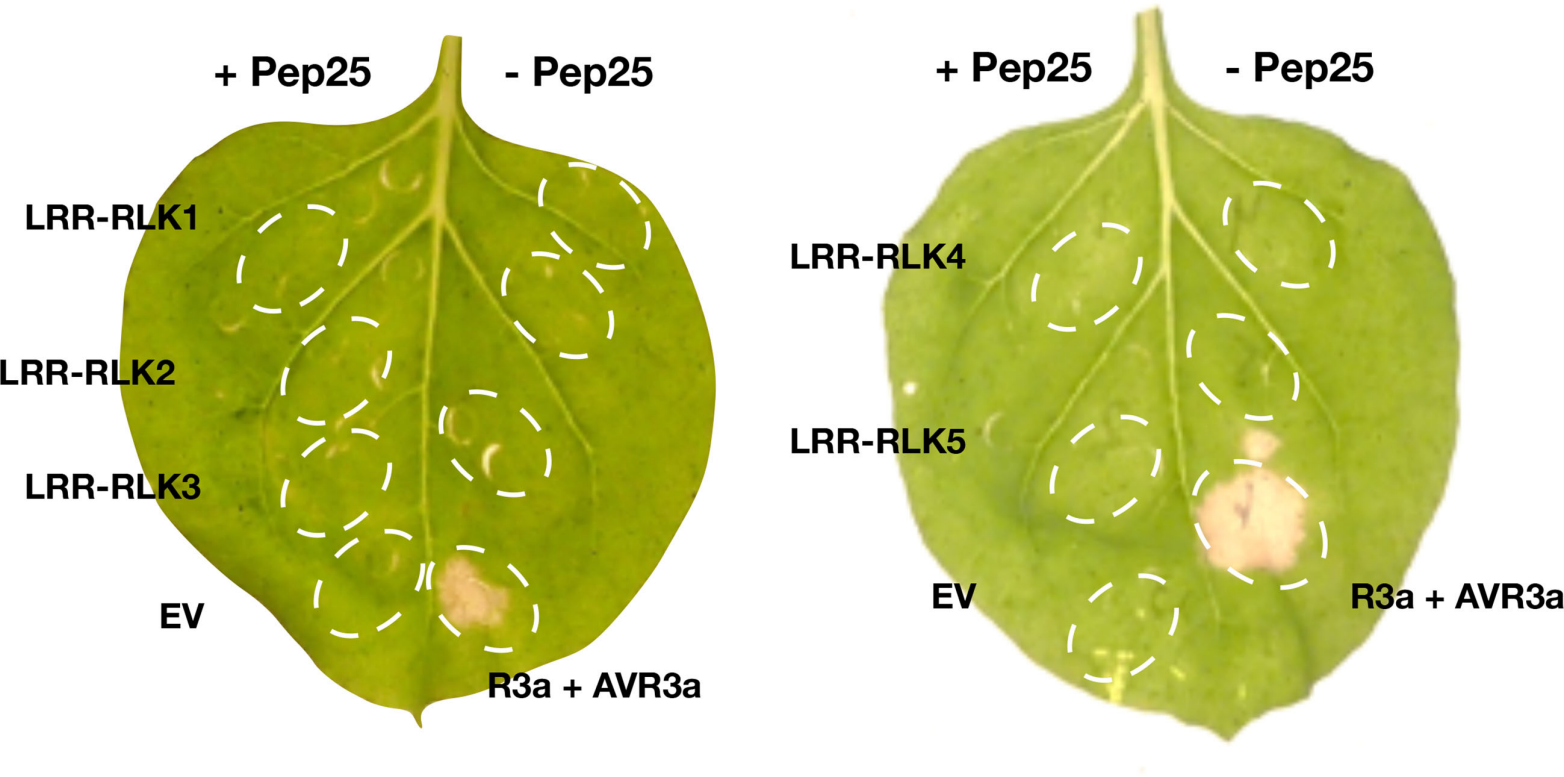
Complementation study of candidate genes. The five candidate genes of the Pep-25 receptor (*LRR-RLK1*, *LRR-RLK2*, *LRR-RLK3*, *LRR-RLK4*, *LRR-RLK5*) were agroinfiltated in *N. benthamiana* leaves, OD_600_ = 1.0, and Pep-25 peptides (2 μM) were infiltrated 2 days later. The photos were taken 3 days after the peptides infiltration. Co-agroinfiltration of *R3a* and *AVR3a* were used as a positive control, empty vector and Pep-25 were used as a negative control.

The genes in the BAC contig (**Figure 3D**) were annotated, many leucine-rich repeat receptor-like kinase (*LRR*-*RLK*) genes locate in this region, and they mainly belong to two *FLS2* and *MDIS2* clades (**Figure S2**). In this mapping interval, the gene architecture of the DM (v4.03) genome is similar as the GIG BAC contig. To obtain the expression profile of these candidate genes, we mapped the RNA-seq reads from the parental lines to the BAC sequence, and five *LRR-RLK* genes (*LRR-RLK1, LRR-RLK2*, *LRR-RLK3*, *LRR-RLK4*, and *LRR-RLK5*) (**Figure 3D**) were up-regulated after *P. infestans* inoculation, and we considered these as candidate genes. We cloned the five *LRR-RLK* genes into overexpression vectors with 35S promoter and transformed them into *Agrobacterium tumefaciens* strain AGL1, these constructs were transiently expressed in *N. benthamiana* leaves followed by Pep-25 infiltration. However, none of the five tested candidate *RLK* genes is responsible for Pep-25 responsiveness (**Figure 4**). Therefore, we speculate that the Pep-25 receptor likely locates in the un-sequenced region of the mapping interval.

## Discussion

Surface immune receptors, such as the receptor of Pep13/25, are believed to confer broad-spectrum and more durable resistance against *Phytophthora* pathogens by recognizing conserved MAMPs, in line with earlier findings with ELR and RLP23 (Albert et al., 2015; Du et al., 2015). The conserved peptides Pep-13/25 are well studied MAMPs (Brunner et al., 2002), and the TGase activity of the *Phytophthora* GP42 protein suggests that this protein is important to the fitness of these pathogens. The Pep-13/25 induced immune responses were reported in the potato cultivar Désirée and parsley cell cultures in the past (Nürnberger et al., 1994; Brunner et al., 2002; Halim et al., 2004), and a potential Pep-13 receptor in parsley was suggested to be a monomeric 100 kDa protein on the plasma membrane (Nurnberger et al., 1995; Nennstiel et al., 1998). However, the gene encoding the Pep-13 receptor in parsley or potato has remained unknown to date.

This study aimed to gain a better understanding of Pep-13/25 triggered immunity in a broad range of plant species and we performed a large-scale investigation on Solanaceae, including wild and cultivated potatoes, tomato, eggplant, pepper, and *Nicotiana* genotypes. Surprisingly, we found the Pep-13/25-induced cell death limited to tuber-bearing *Solanum* species (**Figure 1**), but not in other Solanaceae, which suggests that a Pep-13/25 receptor might have evolved independently in the ancestor of potato. The cell-death phenotype is restricted to potatoes as well. In sum, our data suggest that Pep-13 and Pep-25 recognition capacity from parsley and potato might have evolved independently, however, we cannot rule out that homologues of an ancient Pep-13/25 receptor are present in many different plant species.

We mapped the candidate Pep-25 receptor to a small interval (~0.081 cM) on the top of chromosome 3 in an *RLK* cluster that contains at least 15 full-length or partial *RLK* genes. Five candidate *RLK* genes were cloned and tested transiently in *N. benthamiana*, but none of them gave cell death activity with Pep-13/25, however, we cannot rule out that *N. benthamiana* lacks downstream signalling components for Pep-13/25 recognition. Unfortunately, we were unable to isolate more BAC clones to cover the whole mapping interval, but nonetheless, this work lay the groundwork for the cloning of Pep-13/25 receptor from potato.

Wild tuber-bearing *Solanum* species are renowned as a great resource of resistance genes encoding NLR immune receptors that can be detected by expressing avirulence genes in plants, so-called effectoromics screens. These wild *Solanum* accessions show higher frequency of responses to *Avr* genes than potato cultivars, yet. In contrast, the response to Pep-13/25 was much more prominent in cultivars (*S. tuberosum* Group Tuberosum) and landraces (*S. tuberosum* Group andigena, *S. tuberosum* Group Phureja and *S. stenotomum*) (**Figure 1**). This suggests that the Pep-13/25 receptor may have evolved from an ancestor of the *S. tuberosum* clade and then was selected during potato domestication. However, most potato cultivars are not resistant to *P. infestans*, why it was kept during selection? One possible explanation could be that *P. infestans* has evolved effectors which inhibit Pep13/25 receptor-triggered immunity. Similar findings are reported the a well-studied *P. infestans* RXLR effector Avr3a^KI^ that can inhibit cell death response induced by INF1 (Bos et al., 2006). Alternatively, the Pep-13/25 receptor might be closely linked to domestication traits or has other unknown functions which are important for potato cultivars.

Recently, mutual potentiation of PTI and ETI was reported (Ngou et al., 2021; Yuan et al., 2021), therefore, we can argue that combining the PRR, such as the Pep-13/25 receptor, with *NLR* genes from wild potatoes might help to achieve stronger and more durable resistance against oomycete pathogens. Thus, it remains important to identify PRR that recognize *Phytophthora*, and tools to efficiently identify these genes in the genetically complicated *Solanum* germplasm are most welcome. In this study, we also developed a pipeline to swiftly map potato genes by BSR-Seq (**Figure 2**). It is the first time that BSR-Seq was applied to potato, and our result demonstrated that this approach can dramatically accelerates the speed of map-based cloning. Once the segregating population is generated, the BSR-Seq can be used to rapidly map the gene in weeks. It is noteworthy that all the markers needed for fine mapping can be developed directly based in the filtered SNPs. Meantime, the differential expression data would also facilitate to narrow down the list of candidate genes.

In summary, this work reveals that the receptor of Pep-13/25 is widely distributed in wild and cultivated potatoes. Our development of a BSR-Seq pipeline for rapid mapping of potato genes, and subsequent fine-mapping of Pep-25 receptor to the *RLK* locus is an important step towards the identification of Pep-13/25 receptor in wild and cultivated potatoes.

## Data availability statement

The data presented in the study are deposited in the NCBI SRA and GenBank repository, project number PRJNA893349; accession number OP716690.

## Author contributions

XL and VV conceived and designed the project. XL and YT wrote the first draft with inputs from all the authors. VV and EJ reviewed and edited the manuscript. XL, YT, HF, LD, LR, ED, CA-G, and AK performed the experiments. XL performed the bioinformatics analyses. RV, TN, and VV contributed resources. All authors contributed to the article and approved the submitted version.

## Supplementary material

Supplementary Figure 1 Pep-13/25 are conserved patterns among oomycete pathogens. **(A)** The structure of the cell-wall associated transglutaminases (TGases) GP42 from *Phytophthora sojae* is visualized by web3dmol (Reiss et al., 2011). The Pep-13 peptides are highlighted by cyan (PDB: 3TW5). **(B)** Alignment of Pep-13 and Pep-25 peptides from 8 *Phytophthora* species.

Supplementary Figure 2 Phylogeny of potato LRR-RLKs and the candidate proteins The kinase domains of 365 potato RLK proteins, 12 candidate RLK proteins from GIG362-6 and 21 known RLK proteins were included (blue). I3 from tomato was used as an outgroup The candidate RLK proteins from *Solanum microdontum* subsp. *gigantophyllum* are highlighted by red. The scale bar indicates the number of amino acid substitutions per site.

Supplementary Figure 3 Fine mapping of Pep-25 receptor The molecular markers were designed based on the potato reference genome (DM v4.03), the physical positions (Mb) of the markers on Chromosome 3 are shown in the first line. The polymorphism of each SNPs is shown by “NR” (MCD360-1 allele) or “R” (3341-15 allele). The IDs of recombinants are shown on the left. Pep-25 responsiveness phenotype are noted by “NR” (non-responsiveness) or “R” (responsiveness). The haploid from responsive parent (3341-15) are highlighted by red, and the haploid from the non-responsive parent (MCD360-1) are highlighted by blue.
