# Supplementary figures and images for: Recognition of Pep-13/25 MAMPs of Phytophthora localizes to an RLK locus in Solanum microdontum
Source: Front Plant Sci. 2023 Jan 12;13:1037030. doi: 10.3389/fpls.2022.1037030 (PMC9879208; doi:10.3389/fpls.2022.1037030)

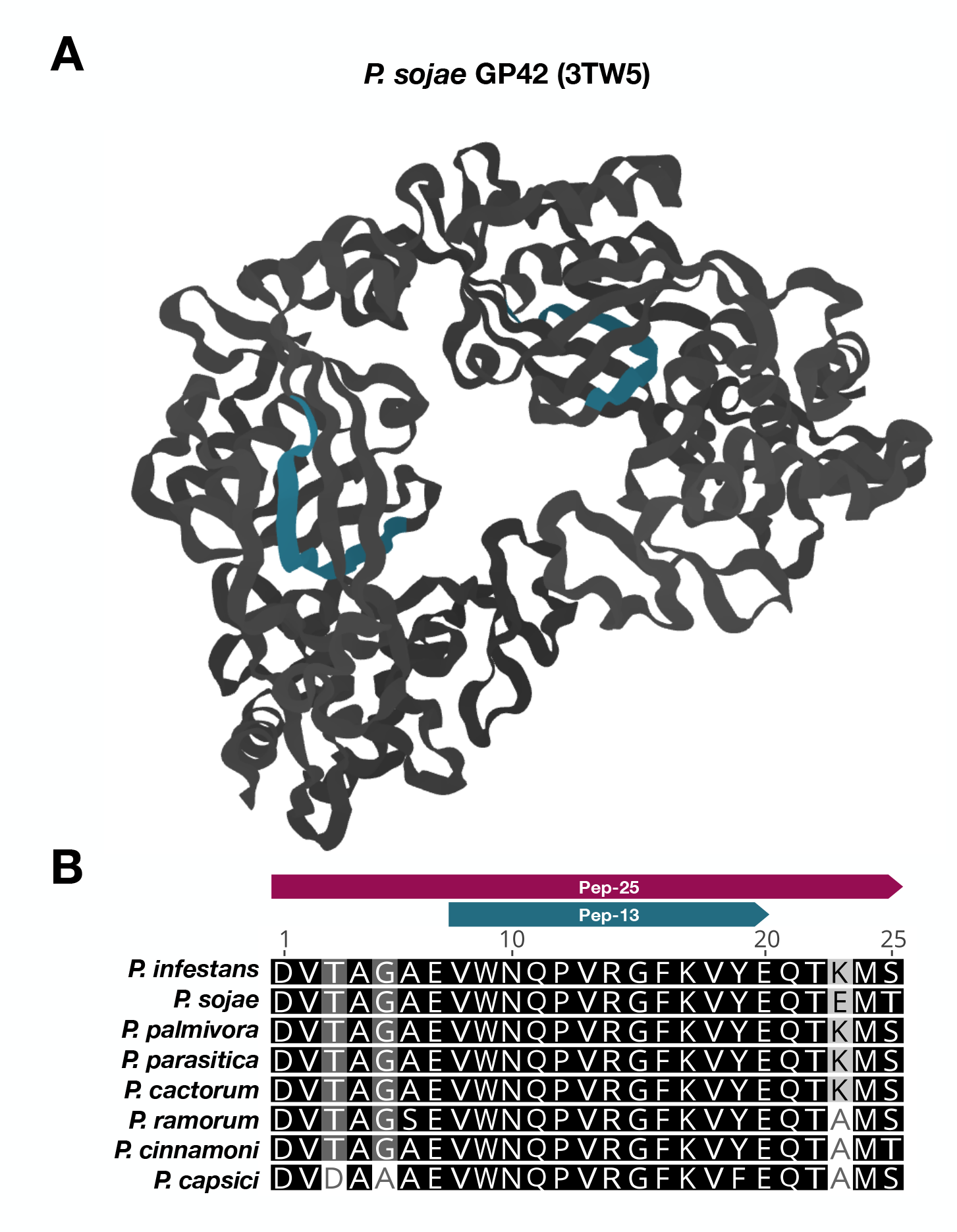

Supplement: Supplementary Figure 1 — Pep-13/25 are conserved patterns among oomycete pathogens. (A) The structure of the cell-wall associated transglutaminases (TGases) GP42 from Phytophthora sojae is visualized by web3dmol (Reiss et al., 2011). The Pep-13 peptides are highlighted by cyan (PDB: 3TW5). (B) Alignment of Pep-13 and Pep-25 peptides from 8 Phytophthora species. [file Image_1.tif]

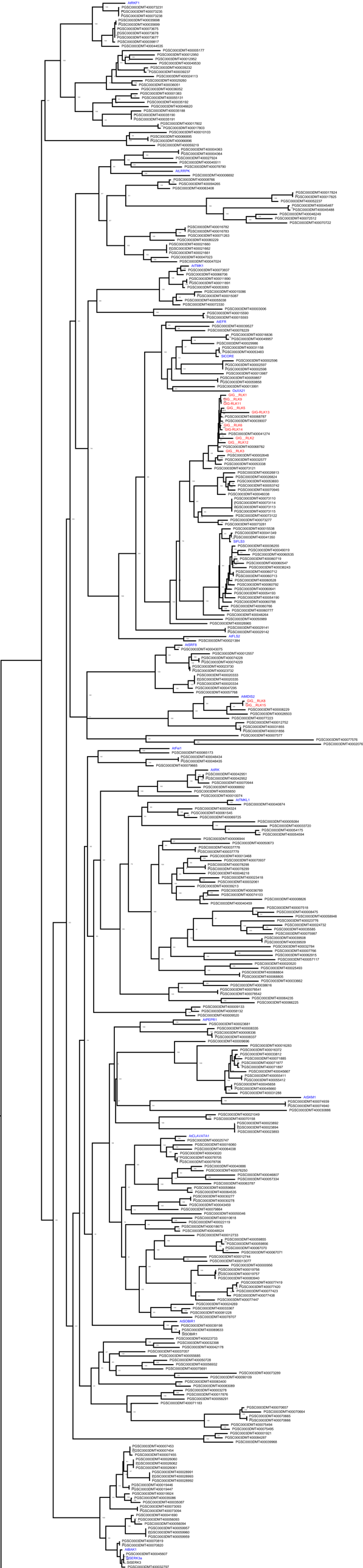

Supplement: Supplementary Figure 2 — Phylogeny of potato LRR-RLKs and the candidate proteins The kinase domains of 365 potato RLK proteins, 12 candidate RLK proteins from GIG362-6 and 21 known RLK proteins were included (blue). I3 from tomato was used as an outgroup The candidate RLK proteins from Solanum microdontum subsp. gigantophyllum are highlighted by red. The scale bar indicates the number of amino acid substitutions per site. [file DataSheet_1.pdf]
