# Supplementary material for: Recognition of Pep-13/25 MAMPs of Phytophthora localizes to an RLK locus in Solanum microdontum
Source: Front Plant Sci. 2023 Jan 12;13:1037030. doi: 10.3389/fpls.2022.1037030 (PMC9879208; doi:10.3389/fpls.2022.1037030)
Supplement: Supplementary Figure 3 — Fine mapping of Pep-25 receptor The molecular markers were designed based on the potato reference genome (DM v4.03), the physical positions (Mb) of the markers on Chromosome 3 are shown in the first line. The polymorphism of each SNPs is shown by “NR” (MCD360-1 allele) or “R” (3341-15 allele). The IDs of recombinants are shown on the left. Pep-25 responsiveness phenotype are noted by “NR” (non-responsiveness) or “R” (responsiveness). The haploid from responsive parent (3341-15) are highlighted by red, and the haploid from the non-responsive parent (MCD360-1) are highlighted by blue. [file DataSheet_2.pdf]

Physical position on Chromosome 3 of DM v4.03 genome

Progeny	0.746	1.22	1.23	1.37	1.429	1.431	Pep-25	1.528 (M63)	1.541 (M66)	1.548	1.560	1.579	1.783_2
3341-15	R	R	R	R	R	R	R	R	R	R	R	R	R
MCD360-1	NR	NR	NR	NR	NR	NR	NR	NR	NR	NR	NR	NR	NR
P18-43	R	R	R	R	R	R	R	R	R	R	NR	NR	NR
3521-1489	R	R	R	R	R	R	R	NR	NR	NR	NR	NR	NR
P22-59	R	R	R	R	R	NR	NR	NR	NR	NR	NR	NR	NR
325	R	R	R	R	NR	NR	NR	NR	NR	NR	NR	NR	NR
P18-41	NR	NR	NR	NR	NR	NR	NR	NR	NR	NR	NR	NR	R
P12-26	NR	NR	NR	NR	NR	NR	NR	NR	NR	NR	NR	NR	R
P21-80	NR	NR	NR	NR	NR	R	R	R	R	R	R	R	R
P16-72	NR	NR	NR	NR	R	R	R	R	R	R	R	R	R
P15-81	NR	NR	NR	NR	R	R	R	R	R	R	R	R	R
3521-56	NR	NR	NR	NR	R	R	R	R	R	R	R	R	R
REC 59	NR	R	R	R	R	R	R	R	R	R	R	R	R
P12-22	NR	R	R	R	R	R	R	R	R	R	R	R	R
P20-43	NR	R	R	R	R	R	R	R	R	R	R	R	R
